# Search for Time Reversal Violating Effects: R-Correlation Measurement in Neutron Decay

**DOI:** 10.6028/jres.110.071

**Published:** 2005-08-01

**Authors:** K. Bodek, G. Ban, M. Beck, A. Bialek, T. Bryś, A. Czarnecki, W. Fetscher, P. Gorel, K. Kirch, St. Kistryn, A. Kozela, M. Kuźniak, A. Lindroth, O. Naviliat-Cuncic, J. Pulut, A. Serebrov, N. Severijns, E. Stephan, J. Zejma

**Affiliations:** Institute of Physics, Jagiellonian University, Cracow, Poland; Laboratoire de Physique Corpusculaire, Caen, France; Catholic University, Leuven, Belgium; Institute of Nuclear Physics, Cracow, Poland; Institute of Physics, Jagiellonian University, Cracow, Poland; Paul Scherrer Institute, Villigen, Switzerland; University of Alberta, Edmonton, Canada; Institute of Particle Physics, ETH, Zürich, Switzerland; Paul Scherrer Institute, Villigen, Switzerland; Laboratoire de Physique Corpusculaire, Caen, France; Paul Scherrer Institute, Villigen, Switzerland; Institute of Physics, Jagiellonian University, Cracow, Poland; Institute of Nuclear Physics, Cracow, Poland; Institute of Physics, Jagiellonian University, Cracow, Poland; Catholic University, Leuven, Belgium; Laboratoire de Physique Corpusculaire, Caen, France; Institute of Physics, Jagiellonian University, Cracow, PolandPaul Scherrer Institute, Villigen, SwitzerlandCatholic University, Leuven, Belgium; St. Petersburg Nuclear Physics Institute, Gatchina, Russia; Catholic University, Leuven, Belgium; University of Silesia, Katowice, Poland; Institute of Physics, Jagiellonian University, Cracow, Poland

**Keywords:** neutron beta decay, time reversal violation

## Abstract

An experiment aiming at the simultaneous determination of both transversal polarization components of electrons emitted in the decay of free neutrons begins data taking using the polarized cold neutron beam (FUNSPIN) from the Swiss Neutron Spallation Source (SINQ) at the Paul-Scherrer Institute, Villigen. A non-zero value of *R* due to the e^−^ polarization component, which is perpendicular to the plane spanned by the spin of the decaying neutron and the electron momentum, would signal a violation of time reversal symmetry and thus physics beyond the Standard Model. Present status of the project and the results from analysis of the first data sample will be discussed.

## 1. Introduction

According to well known theoretical conjectures, supported by experimental observations, the combined charge conjugation and parity symmetry (CP) and time reversal symmetry (T) are closely related by the CPT-theorem. There are two unambiguous pieces of evidence for CP- and T-violation: the forbidden decay modes of neutral *K* and *B* mesons and the excess of the baryonic matter over antimatter in the present universe. However, the CP-violation found in kaon decays, and incorporated into the Standard Model (SM) via the quark mixing mechanism, is too weak to explain the excess of baryons over antibaryons. Therefore, cosmology provides a hint for the existence of an unknown source of T-violation, which is not included in the SM.

The SM predictions of T-violation, originating from the quark mixing scheme, for systems built up of *u* and *d* quarks, are by 7 to 10 orders of magnitude lower than the experimental accuracy available at present. This applies to determinations of the T-violating electric dipole moments as well as to T-violating correlations in decay or scattering processes. With such a strong suppression of the SM contribution these experiments are regarded as important searches for *“Physics beyond the Standard Model”*. It is a general presumption that time reversal phenomena are caused by a tiny admixture of exotic interaction terms. Therefore, weak decays provide a favorable testing ground in a search for such feeble forces. Physics with very slow, polarized neutrons has a great potential in this respect. Our experiment looks for small deviations from the SM in two observables that have never before been addressed experimentally in neutron decay.

## 2. Angular Correlations in *β*-Decay

Direct, i.e., first-order access to the T-violating part of the weak interaction coupling constants, is provided by measurements of directional correlations between the spins and momenta of particles or nuclei involved in the decay process. The lowest order T-violating combination of spins and momenta appears in the form of the mixed triple product. From the experimentally accessible quantities, four triple products can be formed: *R* ***J*** · (***p*** × ***s***), *D* ***J*** · (***p*** × ***p****_ν_*), *V* ***J*** · (***P*** × ***s***), *L* ***P*** · (***p*** × ***s***), where ***J*** is the spin of the parent system, ***s***, ***p*** are the spin and momentum of the detected lepton, ***P*** denotes the momentum of the recoil system and ***p****_ν_* stands for the momentum of the unobserved neutrino. The only system for which both *D* and *R* have been determined is ^19^Ne [2]. The latter two correlations, which require two difficult measurements simultaneously, were not addressed experimentally yet.

For our discussion, the relevant terms in the formula for the decay rate *W* for a semileptonic transition from an oriented sample of nuclei or particles with vector polarization ***J*** can be written as [1]:

$$\begin{array}{l} W\propto \\ \left[1+A\frac{J\cdot p}{E}+B\frac{J\cdot p_{v}}{E_{v}}+D\frac{J\cdot (p\times p_{v})}{EE_{v}}+R\frac{J\cdot (p\times s)}{E}+NJ\cdot s+\cdots \right], \end{array}$$

where *E*, *E_ν_* are the total energies of emitted leptons, and *A* and *B* are the usual decay asymmetry parameters arising from parity violation for the charged lepton and the neutrino, respectively.

### 2.1 The *R*-Correlation

**Fig. 1:**
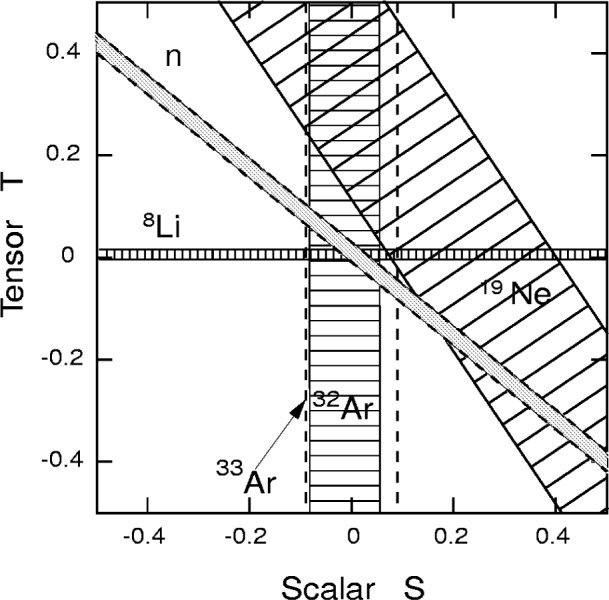
Results from the experiments testing the scalar and tensor weak interaction. The bands indicate ±1s limits. Constraints from the study of the R-correlation in the free neutron decay with an accuracy of ±0.005 are attached. This prediction is arbitrarily fixed at S,T = 0.

The numerical value of the *R*-coefficient represents the transverse component of the electron polarization which is contained in the plane perpendicular to the neutron spin axis. In contrast to *D*, which is sensitive primarily to the complex terms in the vector/axial-vector interference, the P-odd, T-odd *R*-observable may disclose the exotic scalar or tensor interaction terms. The explicit expression for the *R*-amplitude, in terms of Fermi and Gamov-Teller matrix elements *M*_F_, *M*_GT_ and weak interaction coupling constants *C_i_* (*i* = *S*,*V*, *A*,*T*), is given by [1]. For neutron decay, we obtain:

$$\begin{array}{c} R=\frac{T[(C_{V}^{*}+2C_{A}^{*})(C_{T}+C_{T}^{\prime })+C_{A}^{*}(C_{S}+C_{S}^{\prime })]}{{\left|C_{V}\right|}^{2}+3{\left|C_{A}\right|}^{2}}\\ +R_{\text{FSI}}=0.28\cdot S+0.33\cdot T+R_{\text{FSI}}, \end{array}$$

where $S\equiv T[(C_{S}+C_{S}^{\prime })/C_{A}]$ and $T\equiv T[(C_{T}+C_{T}^{\prime })/C_{A}]$ and *M*_F_ = 1, $M_{\text{GT}}=\sqrt{3}$, $C_{V}=C_{V}^{\prime }=ℛC_{V}=1$, $C_{A}=C_{A}^{\prime }=ℛC_{A}=-1.26$, and |*C_S_*|, $\left|C_{S}^{\prime }\right|$, |*C_T_*|, $\left|C_{T}^{\prime }\right|$ ≪ 1 were assumed. While the lowest order expression of *R* vanishes for the SM, the value including final-state interaction becomes finite, *R*_SM_ ≈ 0.001, which is beyond the scope of this experiment, though the value of this correction is known with the absolute precision of 10^−5^ [3]. The exclusion plot in the *S*–*T* plane, including the results from Refs. [2,4] and from electron-neutrino angular correlations in the decay of ^33^Ar [5] is shown in Fig. 1. We note that the neutron experiment, with an accuracy of 0.005 in the *R*-coefficient, has a potential either to determine finite values of the T-violating charged current scalar couplings or to bring a significant improvement in their upper limit.

### 2.2 The *N*-Correlation

Similarly to the *R*-correlation, *N* can be determined by measuring the neutron polarization, and the momentum and transverse polarization of the emitted electron. The experimental apparatus capable of measuring *R* will in a natural way measure *N* simultaneously. The numerical value of the *N*-coefficient multiplied by sin*θ*_e_, *θ*_e_ being the electron emission angle with respect to the neutron spin direction, represents the transverse component of the electron polarization which is contained in the plane spanned by the neutron polarization and the electron momentum. *N* conserves T and is given in Ref. [1]. We note that the Standard Model value of *N* scales with the decay asymmetry *A*, corresponding to:

$$N_{\text{SM}}=-\frac{m}{E}A_{\text{SM}}=\frac{m}{E}\frac{2(\lambda^{2}+\lambda )}{1+3\lambda^{2}}\approx +0.119\frac{m}{E},$$

where *λ* denotes the ratio *C_A_/C_V_*. This neutron decay experiment aims at an absolute sensitivity of 0.5 % which translates into a measurement of *N* at the 5 % (relative) level.

## 3. Experiment

The main challenge of the experiment is the measurement of the polarization of the low energy electrons (end-point energy of 783 keV in neutron decay). Large angle Mott scattering is sensitive to the transverse component of the electron polarization and the analyzing power reaches exceptionally high values of −0.4 to −0.5 [6]. Such a high analyzing power, together with the large polarization of the cold neutron beam (≈90 %) provides an unprecedented sensitivity for spin observables. However, for neutron decay, the difficulty arises from relatively weak decay source (10^3^ – 10^4^ s^−1^). This should be considered in the context of high background generated by slow neutrons captured in the neighborhood of the experiment.

**Fig. 2:**
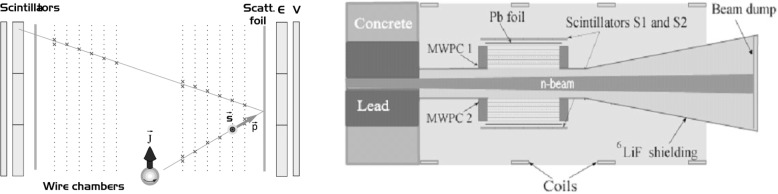
Principle of the measurement (left panel) and the top view of the experimental setup with two detecting systems (right panel). A 1 mg/cm2 thick Pb foil is used as the Mott target.

The principle of the measurement is sketched in Fig. 2. The electron emitted from a polarized neutron and scattered from an analyzing foil is tracked by a system of two multiwire gas chambers and stops in the plastic scintillator. In this way, all the angular and energy information necessary to determine the momentum of the electron and the Mott scattering asymmetry is provided. For the vertically oriented neutron spin in a simultaneous measurement of *R* and *N* one of the correlations will produce an up-down asymmetry while the other leads to a forward-backward asymmetry. One can now fully appreciate the measurement of *N* as an aid for the *R*-measurement: because the deviation of *N* from its SM-value is expected to be small, *N* provides an ideal, positive-effect calibration of the apparatus.

The experiment is carried out on a polarized cold neutron beam line at the spallation source SINQ described in detail in Ref. [7]. An efficient detector providing good rejection of undesired events is of primary importance. The key method of selecting the true events, where the electron emitted in the neutron decay was scattered from the analyzing foil, is based on the electron identification via energy spectrum and the reconstruction of the scattering vertex.

The detector should have low mass and should be constructed of low *Z* materials. The results of the laboratory investigations of the prototype MWPC are described in detail in Ref. [8]. The experience made by testing this detector in the real environment influenced by the neutron beam led to a construction of the full size detectors with an active area of (50 × 50) cm^2^ (see Fig. 2).

**Fig. 3:**
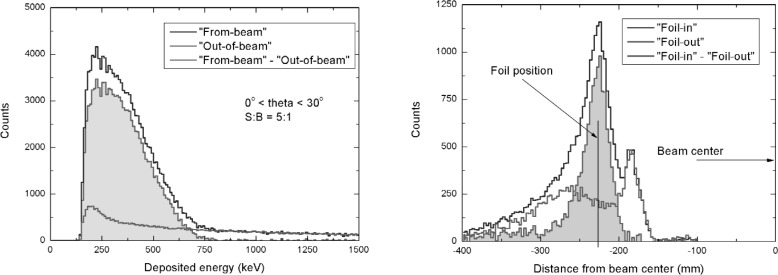
Left panel: Energy spectra of electrons reconstructed as coming from the neutron beam region (“From-beam”) and originating in walls (“Out-of-beam”), respectively. Right panel: Distribution of the reconstructed Mott scattering vertex position. “Foil-in” and “Foil-out” measurement are compared.

The experimental apparatus is complete. Sample data taken during a commissioning run are promising: the electrons originating from neutron decay are clearly identified as can be seen in Fig. 3. The missing part of the experimental *β* spectrum at low energies is due to absorption effects in gas and the energy threshold of the scintillation detectors. Also the distribution of the reconstructed vertex points clearly to the Mott scattering foil position and reveals acceptable background as can be seen from a comparison of “foil-in” and “foil-out” measurements.

It is planned that the experiment will start data taking in summer 2004 and within a few months should collect enough data for the anticipated accuracy of 5 × 10^−3^ for the *R*- and *N*-correlation parameters in the decay of free neutrons.
